# A new concept for risk analysis relating to the degradation of water reservoirs

**DOI:** 10.1007/s11356-018-2634-6

**Published:** 2018-06-29

**Authors:** Krzysztof Boryczko, Lilianna Bartoszek, Piotr Koszelnik, Janusz R. Rak

**Affiliations:** 1 Department of Water Supply and Sewage Disposal, Faculty of Civil and Environmental Engineering and Architecture, Rzeszow University of Technology, Rzeszów, Poland; 2 Department of Environmental Engineering and Chemistry, Faculty of Civil and Environmental Engineering and Architecture, Rzeszów University of Technology, Rzeszów, Poland

**Keywords:** Impact of catchment, Reservoir, Resilience to degradation, Risk, Threat of degradation

## Abstract

This paper presents a proposal for a procedure by which to analyse the risk of reservoirs being degraded. The body of water assessed for its susceptibility to degradation in line with the proposed procedure is Myczkowce Reservoir, SE Poland. This reservoir has a maximum capacity of ten million m^3^ and helps provide hydropower, by serving as a surge tank located above the main Solina Reservoir. On the basis of an assessment of its morphometric and hydrological parameters, Myczkowce Reservoir was assigned to the low-resilience category where risk of degradation was concerned. The primary factors responsible for that are limited capacity in relation to shoreline length, a lack of thermal stratification, and a high value for the Schindler index. These and other environmental parameters provided for Myczkowce’s assignment to the category of susceptible to the impact of matter supplied by its catchment, with this reflecting the instantaneous nature of the basin, high values for the Ohle coefficient, average catchment slope, and the lack of a septic system. The designated risk level supported Myczkowce’s assignment to a category characterised by an “unacceptable” risk of degradation. The proposed method taking two parameters (resilience and susceptibility) into account represents the first universal method for assessing reservoirs without reference to risks such as drought, flooding, or lack of water supply for human consumption. The risk depends only on the reservoir and catchment parameters.

## Introduction

Reservoirs play an important economic and natural role by stabilising hydraulic flows, storing water for drinking, agriculture, etc. and acting as aquatic habitats (Bartoszek and Koszelnik 2016; Kędra et al. 2016). However, specific hydraulic and morphometric parameters leave such waters much more susceptible to degradation than natural lakes (Kozak et al. 2013), ensuring that protection is a key challenge facing water-management bodies. The main cause of a reservoir’s degradation is considered to be an excessive inflow of pollutants from the catchment area, as normally associated with intensive human activity (Bartoszek et al. 2015). This in turn ensures that the most degraded reservoirs of all tend to be those located within heavily urbanised and industrialised catchment areas. However, even reservoirs located in only moderately developed areas can prove to be exposed to major inputs of nutrients and organic matter, as a result of various unfavourable features of the natural environment.

While the biogenic substances of relevance here can originate from both point and non-point sources, a typically unfavourable feature of a catchment area from the pollution point of view would be a large surface area and the presence of slopes, as well as geology conducive to erosion by water (Markowski and Kwidzińska 2015). A high density of rivers also has a marked impact in encouraging rapid transport of matter towards a reservoir. However, adverse environmental conditions characterising a catchment can sometimes be offset by a high resilience to degradation on the part of a reservoir itself. Deep, high-capacity bodies of water are found to degrade much more slowly than shallow ones (Łopata et al. 2013). In the above context, it is very important to interpret the severity of degradation processes appropriately and select restoration methods properly, so as to better reconcile the utility of reservoirs and the nature of their locations. Also important here is the aspect of cost—typically the key factor where the choice of restoration techniques is concerned (Ptak 2017). The EU Water Framework Directive treats reservoirs as bodies of water of unknown characteristics for which interpretation in relation to degradation revolves around similarity to other, more clearly defined ecosystems, such as rivers or lakes. However, as there are no clear algorithms allowing this type of decision-making analysis to be carried out, omissions and excessive economic and environmental costs are both real possibilities.

On the basis of many years of research, Bajkiewicz-Grabowska (1987, 2010) proposed a system allowing for an assessment of, on the one hand, a catchment as a source of matter for a lake, and, on the other, that lake’s resilience to the impact of the catchment. Under this system, resilience is understood as a set of features of a reservoir allowing it to withstand the negative impact of a number of external factors occurring in its basin. In addition to the latter’s morphometric and hydrological parameters and properties, the system takes account of two further factors. First, the Schindler index is calculated as the quotient of the sum of the catchment and reservoir area to its volume, and then, the Ohle coefficient is determined, this being the ratio of the catchment area to the reservoir surface.

While risk analysis can be defined in many different ways, much depends on the relationship with other concepts. Risk assessment, risk characterisation, and risk communication are all included, as is policy relating to risk in the context of what may be of concern to individuals, or to public- and private-sector organisations, or to society as a whole, at the local, regional, national, or global levels.

A useful construct has risk analysis divided into the two components of assessment (identifying, evaluating, and measuring the probability and severity of risks) and management (deciding what to do about those risks) (Haimes 2004; Szpak and Tchorzewska-Cieslak 2015). Risk analysis for technical systems is now highly developed and used i.a. in relation to water supply (Tchórzewska-Cieślak et al. 2012), sewage disposal (Kucharski and Rak 2009), and gas and energy distribution (Tchórzewska-Cieślak et al. 2016). Risk analysis specific to reservoirs in turn takes account of factors such as flooding and low water levels (Wen-Ming et al. 2017; Martona and Kapelan 2014), as well as relevance as a source of drinking water (Yong et al. 2014).

The main aim of this paper has thus been to propose a new concept by which the risk of reservoirs undergoing degradation can be analysed and assessed. A matrix method of analysis is described, with degradation of reservoirs assessed in a proposed risk index comprising the two parameters of (i) susceptibility of the catchment to supply matter to the reservoir and (ii) resilience of the reservoir to degradation.

## The study area

**Fig. 1:**
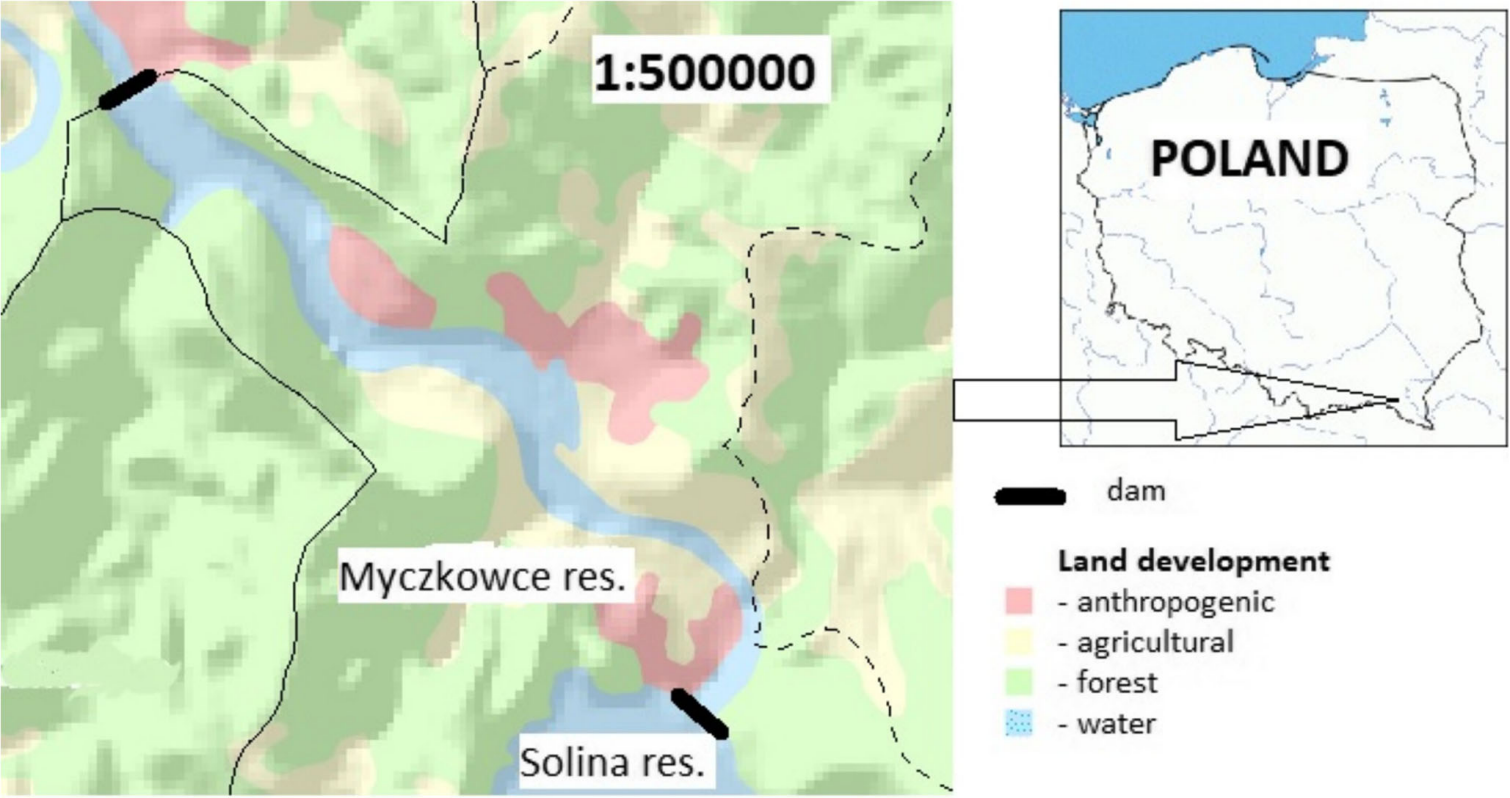
Studied Myczkowce Reservoir (coordinates: 49° 25′ 16″ N, 22° 25′ 27″ E)

Located on the upper part of Poland’s River San, Myczkowce Reservoir came into existence in 1961 (Fig. 1). Together with Solina Reservoir, it forms a cascade which is an element of the Solina–Myczkowce Complex of Hydro-Electric Power Plants—and accounts for about 15% of total water-storage capacity in Poland. Myczkowce Reservoir has a volume of ten million m^3^ and a hydraulic retention time of ca. 4 days (Table 1), while the relevant Solina Reservoir parameters are 470 million m^3^ and 275 days.

**Table 1:** Morphometric parameters of Myczkowce Reservoir, based on Koszelnik (2009)

Parameter	Value
Area [ha]	200
Maximum volume [M m^3^]	10
Average (max.) depth [m]	5 (15)
Hydraulic retention time [days]	4
Length of shoreline [km]	14.2
Total catchment area [km^2^]	1248
Direct catchment area [km^2^]	66

The main alimentation of Myczkowce Reservoir is due to the San, flowing via the waters of the hypolimnion in the upper Solina Reservoir. The outflow through the dam is in fact the reservoir’s main tributary (accounting for over 90% of its water). This situation ensures relatively low temperatures in the reservoir water during summer, but also none of the freezing in winter that would be characteristic where stilling takes place. Moreover, the drainage basin is at the same time a main drainage basin for Solina Reservoir, whose characteristics and influence on water quality have been examined in some detail (Bartoszek and Koszelnik 2016; Koszelnik et al. 2008). The greater part (c. 75%) of the 1250-km^2^ catchment area is taken up by forest, followed by meadows and pastures. Croplands account for only a small fraction of the area. The drainage basin has a low population density of about six inhabitants per square kilometre. A relatively steep (6%) slope favours leaching out of soil and ground cover, especially during periods of intensive atmospheric precipitation and snow melt.

The direct drainage basin of Myczkowce Reservoir is of a mountainous nature and dominated by forest. Such areas are only weakly populated, with buildings concentrated mainly in the southern part, where the villages are situated. However, the Bereźnica and Łobozewski streams can transport municipal wastewaters from localities situated in the upper parts of these two watercourses. Economic activity in these areas is focused on tourism and recreation. Resorts, guest houses, and agritourist farms are located mainly on the outskirts of the reservoir.

## Materials and methods

### Risk analysis

Material risk management involves the three study areas of risk analysis, risk evaluation, and risk control. In turn, risk identification generally means an analysis of risk factors and their sources and a determination of the so-called weak points, as well as consequences (effects) of their occurrence (Kutyłowska 2015).

Analysis concerns the undesirable events that can appear in a system with a certain probability “P” and cause certain losses “C,” which can result in safety failure (Tchórzewska-Cieślak et al. 2014).

From the mathematical point of view, risk (r) is defined (after Apostolakis and Kaplan 1981) as follows:

1 $$ r=P\cdotp C, $$

where

- *P* is a measure of a system’s operating unreliability corresponding with a category of probability, i.e., a frequency.
- *C* is a measure of the consequences corresponding with a category of consequences, i.e., damage expressed in financial units.

Table 2 presents a two-parameter risk matrix, assuming the following risk scales and corresponding point weightings:

- Probability (P): small—1, moderate—2, large—3
- Consequences (C): small—1, moderate—2, large—3

**Table 2:** The two-parameter risk matrix

	*C* = 1	*C* = 2	*C* = 3
*P* = 1	1	2	3
*P* = 2	2	4	6
*P* = 3	3	6	9

In line with this basic matrix for risk assessment, we can analyse different undesirable events assuming the following scale of risk:

- A tolerable risk, where the points range is from 1 to 2
- A controlled risk, where the points range from 3 to 4
- An unacceptable risk, where the points range from 6 to 9

### Susceptibility and resilience to degradation

The natural susceptibility of a body of water to degradation lies in the nature and mode of development of its catchment area, as well as the resilience of the object itself to the latter’s impact. To assess the susceptibility of the Myczkowce Reservoir catchment area to supply it with pollutants, use was made of a modified version of the system proposed by Bajkiewicz-Grabowska (1987, 2010). The systems mentioned have been modified to adapt the risk assessment concerning the reservoirs to progressing degradation.

Each of the evaluated parameters was awarded points on a scale of 1 to 3 (Tables 3 and 4). It was on the basis of the average value in points that the susceptibility of the catchment to supply pollutants (Table 5) and the resilience of the reservoir to such impacts (Table 6) were both determined.

**Table 3:** Assessed susceptibility of the catchment area to supply material to the reservoir, according to the modified system after Bajkiewicz-Grabowska (1987, 2010)

Parameters	Points total		
	1	2	3
Ohle’s coefficient*	< 40	40–150	> 150
Balance type of lake	Exorheic	Endorheic	Flow-through
Density of river network [km km^−2^]	< 1.0	1.0–1.5	> 1.5
Average slope in catchment [‰]	< 10	10–20	> 20
Contribution of endorheic areas [%]	> 45	20–45	< 20
Geological structure of catchment	Clayey, sandy–clayey	Clayey–sandy	Sandy
Land use in catchment	Forest, agricultural–forest, pasture–forest, pasture–agricultural–forest, pasture–agricultural	Agricultural, pasture–forest–agricultural with buildings, forest with buildings	Forest–agricultural with buildings, pasture–agricultural with buildings, agricultural with buildings

*Ratio of total catchment area to reservoir area

**Table 4:** Assessment of the reservoir’s resilience to degradation, in line with the modified system after Bajkiewicz-Grabowska (1987, 2010)

Parameters	Points total		
	1	2	3
Average depth [m]	> 10	5–10	< 5
Ratio of reservoir capacity [‘000 m^3^] to length of shoreline [m]	> 5	3–5	< 3
Participation of water stratification [%]	> 35	20–35	< 20
Ratio of active sediment-layer surface [m^2^] to volume of epilimnion [m^3^]	< 0.10	0.10–0.15	> 0.15
Intensity of water exchange	> 10	5–10	< 5
Schindler’s index* [m^−1^]	< 10	10–30	> 30

*Ratio of the total area of the catchment and reservoir to reservoir volume

**Table 5:** Susceptibility of the catchment area to supply material to the reservoir as compiled on the basis of Bajkiewicz-Grabowska (1987, 2010)

Arithmetic mean	Susceptibility (of catchment)	Description
≤ 1.4	*S* = 1	Matter can reach the reservoir to only a limited degree
1.5–1.9	*S* = 2	Matter can reach the reservoir to a moderate degree
≥ 2.0	*S* = 3	Matter can reach the reservoir to a large degree

**Table 6:** Reservoir resilience to degradation, compiled after Bajkiewicz-Grabowska (1987, 2010)

Arithmetic mean	Resilience (of reservoir)	Description
> 2.4	*R* = 1	Reservoir is resilient to the impact of its catchment to only a limited degree
1.7–2.4	*R* = 2	Reservoir is resilient to the impact of its catchment to a moderate degree
≤ 1.6	*R* = 3	Reservoir is resilient to the impact of its catchment to a large degree

The choice of Myczkowce Reservoir as a research facility was dictated by its status as a medium-sized dam reservoir (of capacity greater than five million m^3^), in which the phenomenon of stratification no longer occurs. At the same time, this reservoir’s catchment is characterised by low anthropogenic impact, albeit with unfavourable features where terrain and size are concerned.

Probability, resilience, and susceptibility are the fundamental characteristics that define the state of any system and are widely used to assess system performance. The concept of resilience was first introduced in ecological systems (Holling 1973) and defined as a measure of the ability of the system to absorb changes and still persist with the same basic structure when subject to stress. Later, widely accepted works (Hashimoto et al. 1982; Meng et al. 2017) saw the application of resilience extended to water-resource systems, with reliability, resilience, and susceptibility reported as potential measures by which the performance of a reservoir system can be evaluated. In (Mereu et al. 2016), the resilience of a reservoir-dominated supply system was assessed in line with current and future changes (in climate, population, tourism, etc.).

In this paper, the authors resigned from the probability parameter given the way factors affecting the risk of degradation of reservoirs mainly occur continuously. Bearing this in mind, the method proposed by the authors lacks the probability parameter and has been adopted as referring solely to the two fundamental parameters of resilience and susceptibility. The specificity of the work described here demands the use of a modified method by which to determine risk; hence, the proposal for its determination is:

2 $$ r=\frac{S}{R} $$

where

- S is the point weighting assigned in line with the susceptibility of the catchment to supply matter to the reservoir, which can assume values of 1 (low catchment susceptibility to supply matter), 2, or 3 (high catchment susceptibility to supply matter).
- R is the point weighting assigned in line with the reservoir’s own resilience to degradation, which can also assume values of 1 (low reservoir’s own resilience to degradation), 2, or 3 (high reservoir’s own resilience to degradation).

Overall, the risk index can assume values as per Table 7.

**Table 7:** The two-parameter risk matrix

	*S* = 1	*S* = 2	*S* = 3
*R* = 1	1	2	3
*R* = 2	$\frac{1}{2}$	1	$\frac{3}{2}$
*R* = 3	$\frac{1}{3}$	$\frac{2}{3}$	1

The risk index can assume values as per Table 7.

In line with the risk matrix, the proposed scale was for:

- A tolerable risk—⟨1/3 ÷ 2/3〉
- A controlled risk—〈1〉
- An unacceptable—⟨3/2 ÷ 3⟩

## Results and discussion

Analysis of the resilience of stagnant water reservoirs to degradation conducted on the basis of their morphometric conditions and susceptibility resulting from the catchment’s influence is used for many ecosystems (e.g., Bartoszek et al. 2015; Markowski and Kwidzińska 2015). For dam reservoirs, which are transient ecosystems between river and lake conditions, it does not always give correct results. While the resilience analysis is usually consistent, the current trophic state does not always correspond to the reality of susceptibility resulting from the nature and supply of pollution from the catchment (Henry et al. 2004; Koszelnik 2009; Mateus et al. 2014). There is therefore a need to develop more precise methods for such analysis. Analysis of the risk commonly used in municipal systems (e.g., Kucharski and Rak 2009; Kutyłowska 2015) allows for the inclusion of a statistical apparatus, which is the probability of occurrence of a specific state of the studied ecosystem. In this way, to the morphometric and hydrochemical factors characterising the susceptibility analysis, an additional aspect is added that allows the analysis of changes that take into account the more sensitive relationships between resilience and degradability.

The evaluation resulted in the reservoir basin being assigned a high (third degree) level of susceptibility (Table 8). In general, the more prone a catchment area is to supply pollutant loads, the greater the risk of a reservoir being exposed to a rapid degradation process. For the purpose of the risk analysis, point weightings for susceptibility and resilience were determined on the basis of measurement parameters, in accordance with Tables 4 and 6:

**Table 8:** Assessment of the susceptibility of the catchment area to supply material to Myczkowce and Solina reservoirs

Parameters	Myczkowce Reservoir		Solina Reservoir^1^	
	Obtained result	No. of points	Obtained result	No. of points
Ohle’s coefficient	624	3	53.4	2
Balance type of lake	Flow-through	3	Flow-through	3
Density of river network [km km^−2^]	0.6	1	0.6	1
Average slope in catchment [‰]	27.8	3	27.0	3
Contribution of endorheic areas [%]	< 20	3	< 20	3
Geological structure of catchment	Sandy–clayey	1	Sandy–clayey	1
Land use in catchment	Pasture–agriculturally–forest	1	Pasture–forest	1
Average points value	2.14		2.0	
Susceptibility of catchment to supply reservoir (S)	3		3	

^1^Data from Bartoszek and Czech (2014)

In line with (2), the level of risk was determined as:

$$ r={}^3/{}_1=3 $$

When the obtained risk value is set against values in the matrix (Table 7), the risk that the reservoir will degrade is found to be “unacceptable.”

While Myczkowce Reservoir has a catchment area that is wooded, only sparsely urbanised and with economic activity mainly focused on tourism, and while it has dominant sandy-loam soils, with lower permeability higher up the slope from the river source to the mouth, conditions do favour the leaching of substances from the ground. In dissolved and suspended form, these are then delivered to the reservoir via mountain streams and rivers characterised by significant decline and high flow dynamics (Zhao and Ding 2016). In turn, assessment of morphometric and hydrological parameters of the reservoir itself determined that Myczkowce Reservoir has only first-degree resilience to the impact due to its catchment area (Table 9).

**Table 9:** The resilience of Myczkowce and Solina reservoirs to degradation

Parameters	Myczkowce Reservoir		Solina Reservoir^1^	
	Obtained result	No. of points	Obtained result	No. of points
Average depth [m]	5	2	22	1
Ratio of reservoir capacity [× 10^3^ m^3^] to length of shoreline [m]	0.70	3	3.35	2
Participation in water stratification [%]	< 20	3	74	1
Ratio of active sediment-layer surface [m^2^] to volume of epilimnion [m^3^]	0.2	3	0.035	1
Intensity of water exchange	91.3	1	1.3	3
Schindler’s index [m^−1^]	125	3	2.38	1
Average points value	2.5		1.5	
Resilience of reservoir (R)	1		3	

^1^Data from Bartoszek and Czech (2014)

The limited resilience is in this case due to relatively limited depth and hence a lack of thermal stratification of the water. This ensures active participation of the entire bottom-sediment surface in secondary pollution of the water and thus a release of nutrients previously stored in the sediment. For this reason, the entire bottom surface was categorised as active, while the entire reservoir volume was classed as epilimnion. Continuous mixing of water promotes the circulation of matter, including nitrogen and phosphorus compounds, intensifying phytoplankton growth during the growing season. The small volume of Myczkowce Reservoir in relation to the length of its shoreline and the high Schindler index is further responsible for the low resilience noted (Simonovic and Arunkumar 2016). The volume of the reservoir determines its ability to dilute the pollution inputting from the large catchment area.

With avoidance of the risk of reservoir degradation borne in mind, Myczkowce should be considered in relation to silting or deepening. A significant increase in the depth and capacity of the reservoir might facilitate stratification of the waters, a feature associated with significantly raised resilience to degradation. The basins of the two reservoirs studied here are characterised by a high degree of susceptibility to the supply of matter, though it must be recalled that the basin of Solina Reservoir is also in large part that of Myczkowce Reservoir. The only rational way to reduce the supply of matter to the two reservoirs would seem to be an increase in out-of-outpour areas, i.e., the creation of mid-forest or mid-field bodies of water capable of retaining loads of nutrients transported along with surface runoff.

In the paper by Bartoszek and Czech (2014), the susceptibility to degradation of the stratified Solina Reservoir was assessed in line with the original system developed by Bajkiewicz-Grabowska (1987). These data were used in this work to verify the proposed risk assessment method. As a result of this study (Bartoszek and Czech 2014), Solina Reservoir gained classification in category 1 where resilience to degradation was concerned, with this attesting to a high level of resilience to catchment impact. In addition, the catchment basin of Solina Reservoir was classified into group 3 as regards susceptibility to the supply of matter, characterised by a moderate possibility of the reservoir being affected (Tables 8 and 9). The original system did not allow for an assessment of the risk of degradation of the reservoir. According to the modified system proposed in this work (Tables 8 and 9), the risk of degradation as determined for the stratified Solina Reservoir has:

$$ S=3 $$

$$ R=3 $$

The level of risk determined as *r* = ^3^/_3_ = 1 means a controlled risk.

The analysis thus yielded a controlled risk of degradation in the case of Solina Reservoir. The marked impact of the catchment is contrasted with the high level of natural resilience to degradation of Solina’s dam reservoir, resulting mainly from considerable depth and hence the huge water volume and occurrence of periodic stratification of waters. This is confirmed by analysis of the available literature for this reservoir, where, notwithstanding what is theoretically a high level of resilience to degradation, it has proved possible to observe periodically elevated values for trophic indicators (Gruca-Rokosz et al. 2011).

While Myczkowce Reservoir was constructed to equalise the main Solina Reservoir, from a natural and hydrological point of view, the latter, larger reservoir serves as a pre-reservoir for Myczkowce and therefore acts as a sink for accumulated pollutants (retained) in aquatic organisms and bottom sediments. In the years 2005–2006, on the basis of field research in a 2-year research cycle, loads of nutrients flowing into Myczkowce Reservoir were determined by measuring concentrations and volume of flows via all tributaries. A study conducted by Bartoszek and Koszelnik (2016) showed that ca. 30% of the phosphorus and ca. 15% of the nitrogen supplied to Solina Reservoir were deposited in a single hydrological year. However, in the next year, nutrient loads discharged into the reservoir were higher than those supplied. Despite the role of Solina Reservoir, Myczkowce has been loaded by a hazardous amount of biogenic substances (Table 10), probably on account of loading with hypolimnetic waters enriched in sedimentary matter.

**Table 10:** Real (L_real_), acceptable (L_accep_.), and dangerous (L_dang_.) phosphorus and nitrogen loads (under the Vollenweider hydraulic model) for the Myczkowce Reservoir (Koszelnik 2009)

	Year	L_real_	L_real_	L_accep._	L_dang._	Threat posed to reservoir
		[mg m^−2^ day^−1^]				
Total phosphorus	2005	66.10	83.75	13.81	27.62	L_real_ > L_dang._
	2006	101.40				
Total nitrogen	2005	1730	2632	207.15	414.3	L_real_ > L_dang._
	2006	3533				

The sizes of permissible and dangerous loads of nutrients for Myczkowce Reservoir were determined on the basis of the Vollenweider hydraulic model that predicts nutrient concentrations in lakes based on nutrient loads and hydraulic residence time (Bajkiewicz-Grabowska 2010). In contrast to the static model, this takes account of the rate of exchange in the reservoir, so it should be used in the case of flow lakes and dam reservoirs. In the 2005–2006 period, phosphorus loads delivered to the reservoir (Table 10) exceeded the threshold for the “dangerous” level more than threefold, indicating that the risk of degradation is high. In the case of nitrogen, the loads delivered (Bartoszek and Koszelnik 2016) were over six times above the “dangerous” level.

Despite such major nutrient loading of Myczkowce Reservoir, only small amounts of phytoplankton are usually observed (Koszelnik 2009). This reflects a low temperature of the hypolimnetic water supplied to the reservoir, together with the very short (on average 4 days) hydraulic retention time, neither of which favours the propagation and development of algal cells. Much of the intensive water exchange (91.3 times a year) is beneficial where resilience to degradation is concerned, because much of the load can be exported in outflow and will prove unavailable for planktonic organisms.

However, to the detriment of Myczkowce Reservoir is its use in the generation of hydroelectric power, with all the frequent associated fluctuations in water level that entails. Alternate uncovering and flooding of the littoral zone here ensures only poor development of the emergent macrophytes that can typically act as a biogeochemical barrier assimilating nutrients from surface runoff. In contrast, submerged macrophytes using the rich base of nutrients are well developed. In Myczkowce Reservoir, these cover up to 50% of the littoral zone (Koszelnik 2009), while abundant development of submerged vegetation is characteristic of its clear waters.

Simplicity of interpretation of results speaks for the use of risk assessment methods, these representing a quick and efficient tool by which the degradation of reservoirs can be assessed. Results obtained in the form of a risk value, through the use of a simple two-parameter risk matrix, offer a rapid answer as to whether a risk is tolerable, controlled, or unacceptable. Compared with the Bajkiewicz-Grabowska method, the proposal does not require knowledge of the specific reservoir-catchment relationship, given that each element is assessed separately. Use of the proposed formula for the risk index offers a specific result and a risk category simultaneously.

## Conclusions

Analysis of environmental features of the catchment area parameters characterising Poland’s Myczkowce Reservoir shows that the catchment area is prone to delivering organic and biogenic matter to the body of water, mainly on account of its large area and type of terrain. The reservoir is characterised by only a low level of natural resilience to the degradation the catchment area causes, with decisive influences here being limited depth and the small volume of retained water. A rapid process of degradation is possible in the circumstances of limited resilience to a catchment-area impact characterised by flexibility of supply of material. The method of determining risk of degradation proposed here may prove useful in the assessment and comparison of different reservoirs. The proposed method taking into account two parameters (resilience and susceptibility) is the first universal method for assessing reservoirs that does not extend specifically to risks such as drought, flooding, or lack of water supply for human consumption. Here, risk is taken to depend solely on reservoir and catchment parameters.
